# Long Non-coding RNA Small Nucleolar RNA Host Gene 14, a Promising Biomarker and Therapeutic Target in Malignancy

**DOI:** 10.3389/fcell.2021.746714

**Published:** 2021-09-23

**Authors:** Shen Shen, Yanfang Wang, Yize Zhang, Zihui Dong, Jiyuan Xing

**Affiliations:** ^1^Precision Medicine Center, Gene Hospital of Henan Province, The First Affiliated Hospital of Zhengzhou University, Zhengzhou, China; ^2^Department of Infectious Diseases, The First Affiliated Hospital of Zhengzhou University, Zhengzhou, China; ^3^Department of Pharmacy, The First Affiliated Hospital of Zhengzhou University, Zhengzhou, China

**Keywords:** malignancy, lncRNA, SNHG14, biomarker, prognosis

## Abstract

Small nucleolar RNA host gene 14 (SNHG14) is a long non-coding RNA found to be overexpressed in various types of cancers. Moreover, the expression level of SNHG14 was closely associated with multiple clinicopathological characteristics such as prognosis, tumor differentiation, TNM stage, and lymph node metastasis. Functionally, gain- and loss-of-function of SNHG14 revealed that overexpressed SNHG14 promoted cancer cell viability, invasion, and migration, whereas its down-regulation produced the opposite effect. Mechanistically, regulating its target gene expression by sponging distinct miRNAs might be the major mechanism underlying the oncogenic functions of SNHG14. Thus, SNHG14 might be a promising prognostic biomarker and therapeutic target for cancers. In this review, we discuss the expression profile, biological function, and molecular mechanisms of SNHG14 in cancers to provide a molecular basis for the clinical utility of SNHG14 in the future.

## Introduction

Long non-coding RNAs (lncRNAs) are a group of non-protein-coding RNAs longer than 200 nucleotides in length (Chan and Tay, 2018). Growing evidence shows that lncRNAs play vital roles in normal cellular processes through complicated mechanisms (Ørom et al., 2010; Tian et al., 2010). Dysfunction of lncRNAs implicates pathological conditions, especially cancer (Huarte, 2015; Schmitz et al., 2016; Bhan et al., 2017; Yarani et al., 2018; Zhang and Tang, 2018; Simion et al., 2019). Previous studies have determined the biological function of lncRNAs in cellular development and metabolism, including genome rearrangement (Aznaourova et al., 2020), chromatin modification (Han and Chang, 2015), splicing (Romero-Barrios et al., 2018; Corona-Gomez et al., 2020), mRNA decay (Zhu et al., 2013), genetic imprinting (Barlow and Bartolomei, 2014), and translational regulation (Akhade et al., 2017). Emerging lncRNAs are understood to be involved in regulating gene expression at the transcriptional and post-transcriptional levels (Zhang et al., 2019a). Notably, several lncRNAs usually play an oncogenic role or tumor suppressor roles in carcinogenesis and cancer progression by affecting tumor cell differentiation, viability, invasion, migration, apoptosis, and drug resistance (Wang J. et al., 2018; Ghafouri-Fard et al., 2020a,b).

LncRNA small nucleolar RNA host gene 14 (SNHG14) is a new lncRNA located on chromosome 15q11.2 in humans. It has played an essential role in promoting inflammation microglia activation (Qi et al., 2017; Zhong et al., 2019; Jiang et al., 2021), sepsis-induced acute kidney injury (Shi et al., 2021), and LPS-induced acute kidney injury (Yang et al., 2021). In addition to its function in tumorigenesis and progression, SNHG14 was found to function as a competing endogenous RNA for microRNAs-382-5p (miR-382-5p) to regulate SPIN1 expression in non-small cell lung cancer (Chen et al., 2020). Tang and Yang (2020) reported that SNHG14 binds to miR-656-3p using dual-luciferase reporter assay in hepatocellular carcinoma (HCC). Xie et al. (2020) found that SNHG14 promoted pancreatic ductal adenocarcinoma progression by regulating E-cadherin expression by interacting with EZH2. Collectively, the regulatory mechanisms of SNHG14 are highly complicated and unclear.

In this study, we summarize the latest evidence of SNHG14 in human cancers, especially its abnormal expression, biological functions, and molecular mechanisms, and discuss the potential clinical value of SNHG14 as a novel method for cancer diagnosis, prognosis, and treatment.

## Clinical Relevance of Small Nucleolar RNA Host Gene 14 in Cancers

Tissue-specific expression patterns indicate that lncRNAs might be served as potential biomarkers and provide a rationale to target them clinically (Statello et al., 2021; Winkle et al., 2021). To explore the role of SNHG14 in cancer, some research groups have studied the expression profile, roles, and clinical significance of SNHG14 in various types of cancers (Table 1). Feng et al. (2021) reported that 37 of 62 of SNHG14 expressions were significantly increased in bladder cancer tissues than in normal control tissues. Another study found this lncRNA was overexpressed in HCC tissues and cell lines (Lin et al., 2021). Up-regulation of SNHG14 in tumor tissues was also found in multiple cancer types, such as NSCLC (Zhang et al., 2019d), HCC (Pu et al., 2019), osteosarcoma (Hou and Mao, 2020), ovarian cancer (Zhao and Huang, 2019), glioma (Lu et al., 2020), prostate cancer (Luo et al., 2020), breast cancer (Dong et al., 2018a), clear cell renal cell carcinoma (Liu et al., 2017), retinoblastoma (Sun et al., 2020), acute myeloid leukemia (Wang et al., 2021a), pancreatic cancer (Zhang et al., 2019b), colorectal cancer (Han et al., 2020), cervical cancer (Ji et al., 2019), and endometrial cancer (Zhao et al., 2020). Notably, the expression of SNHG14 in glioma (Wang Q. et al., 2018), colorectal cancer (Zhang W. et al., 2020), and endometrial carcinoma (Zhang K. et al., 2020) was controversial. We suggest that large sample size and cross-regional or even cross-national multi-center large sample verification may provide better evidence for the conclusion. We believe that the existing studies are contradictory and may be affected by various factors such as detection methods, sensitivity and experimental conditions These seemingly contradictory conclusions suggest that further research is needed to this regard.

**TABLE 1:** Expression of SNHG14 in clinical samples.

**Cancer types**	**Numbers of tissues**	**Expression**	**Clinicopathological characteristics**	**Prognosis**	**References**
Bladder cancer	62 pairs	High	Advanced TNM stage, tumor invasion stage, and lymph node metastasis	Poor	Feng et al., 2021
	24 pairs	High	Not studied	Poor	Li et al., 2019
Non-small cell lung cancer	99 pairs	High	Larger tumor size and advanced TNM stage	Poor	Zhang et al., 2019d
	74 pairs	High	Not studied	Not studied	Zhao et al., 2020
	50 pairs	High	Not studied	Not studied	Chen et al., 2020
Hepatocellular carcinoma	55 pairs	High	Later stage	Not studied	Xu X. et al., 2020
	40 pairs	High	Not studied	Poor	Zhang H. et al., 2020
	66 pairs	High	Advanced stage	Poor	Liao et al., 2021
Osteosarcoma	31 pairs	High	Not studied	Not studied	Hou and Mao, 2020
Ovarian cancer	24 pairs	High	Not studied	Poor	Zhao and Huang, 2019
	56 pairs	High	Not studied	Poor	Zhao J. L. et al., 2019
Glioma	8 pairs	High	Not studied	Not studied	Lu et al., 2020
	29 pairs	Low	Not studied	Not studied	Wang Q. et al., 2018
Breast cancer	36 pairs	High	Not studied	Not studied	Dong et al., 2018b
Retinoblastoma	43 pairs	High	Advanced stage and differentiation grade	Poor	Sun et al., 2020
Diffuse large B cell lymphoma	3 pairs	High	Not studied	Not studied	Zhao L. et al., 2019
	21 pairs	High	Not studied	Not studied	Tian et al., 2021
Acute myeloid leukemia	57 pairs	High	Not studied	Not studied	Wang et al., 2021a
Pancreatic cancer	65 tumor and 30 normal tissues	High	Not studied	Not studied	Zhang et al., 2019b
	45 pairs	High	Poor tumor differentiation, advanced TNM stage, and nodal metastasis	Not studied	Deng et al., 2019
	58 pairs	High	Advanced TNM stage and positive lymph node metastasis	Poor	Xie et al., 2020
Colorectal cancer	92 pairs	Low	Not studied	Poor	Zhang W. et al., 2020
	50 pairs	High	Not studied	Not studied	Han et al., 2020
	32 pairs	High	Not studied	Poor	Pei et al., 2019
	30 pairs	High	Tumor stage, tumor size, and distant metastasis	Not studied	Wang et al., 2021b
Cervical cancer	80 pairs	High	Advanced FIGO stage, differentiation, and lymph node metastasis	Poor	Ji et al., 2019
	30 pairs	High	Large tumor size, later stage and a higher incidence of lymph node metastasis	Poor	Zhang et al., 2019c
Endometrial cancer	52 pairs	High	Larger tumor size and distance metastasis	Poor	Zhao et al., 2020
	53 pairs	Low	FIGO stage, histological grade, and lymphatic metastasis	Better	Zhang K. et al., 2020

Importantly, the expression level of SNHG14 has been demonstrated correlated with prognosis in patients with NSCLC (Zhang et al., 2019d), HCC (Zhang H. et al., 2020), ovarian cancer (Zhao J. L. et al., 2019), retinoblastoma (Sun et al., 2020), pancreatic ductal adenocarcinoma (Xie et al., 2020), colorectal cancer (Pei et al., 2019), cervical cancer (Ji et al., 2019), and endometrial cancer (Zhao et al., 2020). Furthermore, some researchers discovered that highly expressed SNHG14 was positively correlated with large tumor size, advanced TNM stage, distant metastasis and poor tumor differentiation in bladder cancer (Feng et al., 2021), NSCLC (Zhang et al., 2019d), HCC (Liao et al., 2021), prostate cancer (Luo et al., 2020), retinoblastoma (Sun et al., 2020), pancreatic cancer (Deng et al., 2019), and cervical cancer (Ji et al., 2019). The results indicate that SNHG14 can become a prognostic indicator of cancers.

ROC curves analysis showed that the sensitivity of SNHG14 in HCC was 98.5% and the optimal cutoff value of SNHG14 was 1.22 (Tang and Yang, 2020). In bladder cancer, the survival curve analysis of SNHG14 showed that the area under the curve was 0.842 and the cutoff value was 2.714, indicating the diagnostic potential of SNHG14 (Li et al., 2019). Zhao et al. (2020) also confirmed the diagnosis efficiency of SNGH14 in ovarian cancer.

Generally, the aforementioned results suggest that SNHG14 plays an oncogenic role in various types of cancer, and it may serve as a new biomarker for cancer diagnosis and prognosis, although further investigation is required for clinical application.

## Biology Function and the Molecular Mechanism of Small Nucleolar RNA Host Gene 14 in Various Cancers

Emerging evidence revealed the expression of SNHG14 in tumor cell lines and the effect of knockdown or overexpression of this lncRNA on tumor cell malignant characteristics such as proliferation, invasion, migration, apoptosis, and drug resistance. The regulatory mechanisms of SNGH14 are complex in distinct types of cancers, even in one cancer. The major mechanism underlying the tumor-promoting function of SNHG14 is to regulate target genes via competing with miRNAs (Table 2). In the following sections, we focus on the function of SNHG14 in various cancers.

**TABLE 2:** Mechanism underlying the function of SNHG14 in various cancers.

**Cancer types**	**Assessed cell lines**	**Function**	**Molecular mechanism**	**Target genes and related signal pathway**	**References**
Bladder cancer (BCa)	BCa cell lines (T24, 5637, UMUC-3, and EJ) and normal bladder epithelial cells SV-HVC-1	Invasion, migration, and proliferation	Sponging miR-211-3p	ESM1	Feng et al., 2021
	Normal bladder transitional epithelial cell line SV-HUC1 and BCa cell lines T24, UC9, PAL19, and UC19	Proliferation	Sponging miR-150-5p	VAMP2	Li et al., 2019
Non-small cell lung cancer (NSCLC)	NSCLC cells A549, NCI-H1975, NCI-H1299, SK-MES-1, and normal human bronchial epithelial 16HBE cells	Proliferation	Sponging miR-340	Not studied	Zhang et al., 2019d
	Bronchial epithelioid cell line 16HBE and two NSCLC cell lines A549 and H1299	Cisplatin resistance	Sponging miR-34a	HMGB1	Jiao et al., 2019
	NSCLC cell line PC9, gefitinib-resistant PC9 cell line (PC9/GR)	Gefitinib resistance	Sponging miR-206-3p	Not studied	Wu et al., 2019
	Normal bronchial epithelial 16HBE cells and two NSCLC cell lines (A549 and SK-MES-1)	Proliferation, invasion, and migration	Sponge for miR-206	Not studied	Zhao et al., 2020
	NSCLC cell (A549) and DDP-resistant NSCLC cell (A549/DDP)	Cisplatin resistance	Sponging miR-133a	HOXB13	Xu L. et al., 2020
	H1299 and A549 cells compared with that in normal lung cell BEAS-2B	Migration, invasion, and apoptosis	Sponging miR-382-5p	SPIN1	Chen et al., 2020
Hepatocellular carcinoma (HCC)	HCC cell lines (Hep3B and Huh-7) and normal liver cell line L02	Proliferation and apoptosis	Sponging miR-4673	SOCS1	Pu et al., 2019
	Liver epithelial cell line (THLE-2) and the HCC cell line (Huh-7, Hep3B)	Cell proliferation and apoptosis	Sponging miR-217-5p	MAPK/ERK signaling	Xu X. et al., 2020
	HCC cells (HepG2, Hep3B, MHCC-97H, and Huh-7) and human hepatocyte cells (LO2)	Proliferation, invasion, and migration	Sponging miR-656-3p	SIRT5	Tang and Yang, 2020
	Human hepatic cell line L02 cells, human HCC cell lines Hep3B, SMMC7721, Huh7, HepG2, and MHCC-97H cells	Proliferation, migration, and angiogenesis	Regulating PABPC1	PTEN signaling pathway	Zhang H. et al., 2020
	Human hepatic cell line L02 cells and human HCC cell lines HepG2, Hep3B, HLF, MHCC-97H	Proliferation, migration, and invasion	Sponging miR-876-5p	SSR2	Liao et al., 2021
Osteosarcoma	143B, MG-63, Saos-2, HOS, and U2OS cell lines and normal human osteoblastic cell line (HFOB1.19)	Proliferation, migration, and invasion	Sponging miR-433-3p	FBXO22	Hou and Mao, 2020
Ovarian cancer	Normal cell line HOSEpiC and ovarian cancer cell lines including C13K, SKOV3, 3AO, and OVCAR3	Proliferation and cell cycle progression	Sponging miR-125a-5p	DHX33	Zhao and Huang, 2019
	A2780, TO-V112D, HO-8910, OVCAR-3, and SKOV3 and one normal ovarian cells (ISOE80)	Migration and invasion	Regulating DGCR8	DGCR8	Zhao J. L. et al., 2019
Glioma	Glioma cell lines U251 and U87 and normal brain glial cell line HEB	Invasion, and apoptosis	Sponging miR-92a-3p	Not studied	Wang Q. et al., 2018
Prostate cancer	Normal human myofibroblast stromal cell (WPMY1) and human prostate cancer cell lines, including LNCaP, 22RV1, PC-3, and DU145	Not studied	Sponging miR-5590-3p	YY1	Luo et al., 2020
Breast cancer	Breast cancer cell lines SKBR-3 and BT474	Trastuzumab resistance, proliferation, and invasion	Regulating expression PABPC1	Not studied	Dong et al., 2018b
Clear cell renal cell carcinoma (ccRCC)	Human ccRCC cell lines A-498, 786-O, Caki-2, and Caki-1 and human normal renal epithelial cell line HK-2	Migration and invasion	Regulating N-WASP	Not studied	Liu et al., 2017
Retinoblastoma (RB)	Three RB cell lines Y79, SO-RB50, and Weri-RB-1 and normal retinal pigmented epithelial ARPE-19 cell line	Proliferation, migration and invasion, and apoptosis	Sponging miR-124	STAT3	Sun et al., 2020
Diffuse large B cell lymphoma	Lymphoblastoid B cell (GM12878), human renal epithelial cell (293T), murine DLBCL cell (A20), and DLBCL cells (OCI-LY7, DB, U2932, and FARAGE)	Proliferation, migration and epithelial-mesenchymal transition	Sponging miR-5590-3p	ZEB1	Zhao L. et al., 2019
	Lymphoblastoid B cell (GM12878), germinal center B cell (GCB)-like cell line (OCI-LY-7), and activated B cell (ABC)-subtype cell line (OCI-LY-3 and RCK-8)	Proliferation, apoptosis, and migration	Sponging miR-152-3p	Not studied	Tian et al., 2021
Acute myeloid leukemia (AML)	Human normal bone marrow CD34^+^ cells and AML cell lines (MV-4–11, AML-193, HL-60, and KG-1 cells)	Proliferation and apoptosis	Sponging miR-193b-3p/MCL1	Not studied	Wang et al., 2021a
Pancreatic cancer	Pancreatic cancer cell line (SW1990), normal pancreatic cell line (HPDE6C7), and the human embryonic kidney 293T cell line	Proliferation, migration, and invasion	Sponging miR-101	Not studied	Zhang et al., 2019b
	Normal immortalized human pancreatic epithelial cell line (HPDE6C7) and four human pancreatic cancer cell lines (CFPAC-1, BXPC3, L3.6pl, and Panc-1)	Proliferative, invasive potentials, and apoptosis	Sponging miR-613	Annexin A2	Deng et al., 2019
Pancreatic ductal adenocarcinoma	Panc1, Panc28, AsPC1, and BxPC3 and a human pancreatic ductal epithelial cell line HPDE	Proliferation and invasion ability	Interacting with EZH2	Not studied	Xie et al., 2020
Colorectal cancer (CRC)	Normal human colorectal cell line NCM460 and five CRC cell lines (LoVo, SW620, SW480, HCT116, and HT-29)	Proliferation, motility, and epithelial–mesenchymal transition	Interacting with EZH2	EPHA7	Di et al., 2019
	Normal human colon epithelial cells (FHC) and other five human CRC cancer cells (Caco-2, HT-29, HCT-116, SW480, a nd SW62)	Cell growth, migration, invasion, and apoptosis	Sponging miR-92b-3p	Not studied	Zhang W. et al., 2020
	CRC cell lines (SW620 and SW480), a normal human colon mucosal epithelial cell line (NCM460), and the human embryonic kidney (HEK) 293 T cell line	Cisplatin resistance, proliferation, migration, and invasion	Sponging miR-186	ATG14	Han et al., 2020
	CRC cell lines SW620, HCT116 cells, and human normal epithelial colonic cells NCM460	Proliferation, migration, invasion, and apoptosis	Sponging miR-944	KRAS/PI3K/AKT pathway	Pei et al., 2019
	CRC cell lines (LoVo, RKO, SW480, and HT-29) and normal colon epithelial cells (NCM460)	Proliferation, metastasis, and epithelial-mesenchymal transition process	Sponging miR-32-5p	SKIL	Ye et al., 2019
			Sponging miR-519b-3p	DDX5	Wang et al., 2021b
	Human CRC cell lines (SW480, HT-29, HCT-8 and DLD-1) and human normal colon epithelial cells (NCM460)	Proliferation, migration, invasion, and apoptosis			
Cervical cancer (CC)	CC cell lines (SiHa, HeLa, C33a, Me180, and Ms751) and human normal cervical cell lines (Ect1/E6E7)	Proliferation, migration, invasion, and apoptosis	Sponging miR-206	YWHAZ	Ji et al., 2019
	CC cell lines (SW756, SiHa, and HeLa) and normal endo-cervical epithelial cell line (End1/E6E7)	Proliferation and apoptosis		JAK-STAT pathway	Zhang et al., 2019c
Endometrial cancer (EC)	Human EC cell lines (HEC-1A, HEC-1B, KLE, and Ishikawa) and human endometrial stromal cell line (T-HESC)	Cell proliferation and apoptosis	Sponging miR-655-3P	Not studied	Zhao et al., 2020
	Human embryonic stem cell and EC cell lines (HEC1-A, HEC1-B, AN3CA, and Ishikawa)	Viability, migration, and invasion	Sponging miR-93-5p	Not studied	Zhang K. et al., 2020

### Bladder Cancer

Bladder cancer is one of the most common cancers and has high mortality worldwide (DeGeorge et al., 2017; Lenis et al., 2020). Recently, lncRNAs were found to be closely correlated with bladder occurrence and development (Martens-Uzunova et al., 2014; Cao et al., 2020; Li et al., 2020). However, the molecular mechanism of lncRNAs in the pathogenesis of bladder cancer is still unclear. The expression level of SNHG14 in bladder cancer cell lines (T24, 5637, UMUC-3, and EJ) was higher than in normal bladder epithelial cells SV-HCV-1 (Li et al., 2019; Feng et al., 2021). Feng et al. (2021) reported that knockdown of lncRNA in T24 cells suppressed cell proliferation, migration, and invasion, while facilitating cell apoptosis, overexpression this lncRNA shows an opposite effect. The results of bioinformatic analysis and luciferase reporter assay demonstrated that SNHG14 functions as a cancer-promoting gene by targeting miR-211-3p to regulate ESM1 expression (Feng et al., 2021). In other studies, the overexpression of SNHG14 was found to accelerate the proliferative potential by sponging miR-150-5p to degrade VAMP2 expression (Li et al., 2019). The aforementioned results suggest that SNHG14 is a potential therapeutic target for bladder cancer.

### Non-small Cell Lung Cancer

Lung cancer is the most commonly diagnosed cancer, with approximately 1.8 million cancer-related deaths worldwide in 2018 (Bray et al., 2018). NSCLC accounts for 85% of all lung cancer cases (Ginn et al., 2020). The 5-year survival rate for patients with advanced-stage NSCLC remains approximately 14% (Ko et al., 2018). This highlights a need to develop new ways to tackle the disease. Zhang et al. (2019d) reported that SNHG14 expression was markedly higher in NSCLC cell lines (including A549, NCI-H1975, NCI-H1299, and SK-MES-1) than normal 16HBGE cells, and overexpression of SNHG14 promoted cell proliferation by targeting miR-340. A recent study found that overexpression of SNHG14 facilitates NSCLC cell proliferation, invasion, and migration by regulating G6PD expression by sponging miR-206 (Zhao et al., 2020). Similarly, Chen et al. (2020) demonstrated that SNHG14 accelerated NSCLC progression via the miR-382-5P/SPIN1 axis. Additionally, SNHG14 was reported to influence NSCLC cisplatin resistance by modulating the HMGB1 expression through targeting miR-34a (Jiao et al., 2019). Specifically, in this research, the expression of SNHG14 was remarkably high in cisplatin-resistant NSCLC cell lines (A549 and H1299) compared with that in the human bronchial epithelioid cell line (16HBE). SNHG14 silencing or overexpression miR-34a promoted cell sensitivity to cisplatin (Jiao et al., 2019). SNHG14 was also revealed to regulate the cisplatin resistance through the miR-133a/HOXB13 pathway (Xu L. et al., 2020). Wu et al. (2019) found that SNHG14 expression was increased in gefitinib-resistant cells, and overexpression of SNGH14 promoted gefitinib resistance by facilitating cell growth and restraining cell apoptosis through interacting with miR-206-3P.

**FIGURE 1:**
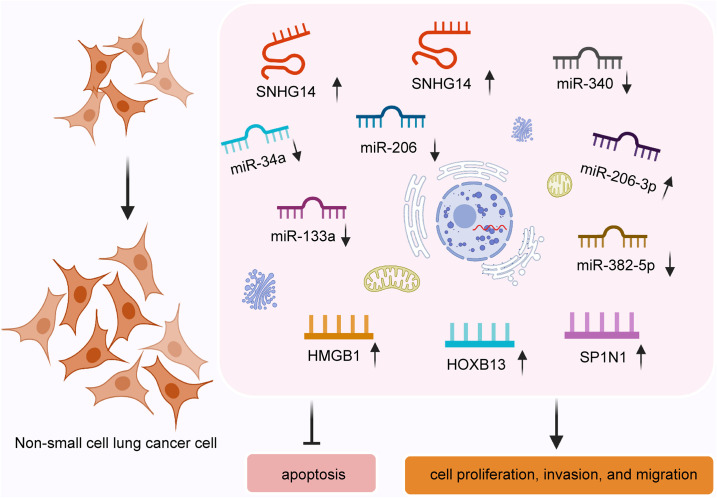
SNHG14 enhances non-small cell lung cancer cell proliferation, invasion, and migration and inhibits cell apoptosis by regulating target genes through sponging miRNAs.

Briefly, SNHG14 plays a vital role in promoting NSCLC cell proliferation, migration, invasion, and chemoresistance and inhibiting cell apoptosis by regulating targets by sponging different miRNAs (Figure 1). SNHG14 is expected to provide a novel strategy for NSCLC treatment.

### Hepatocellular Carcinoma

**FIGURE 2:**
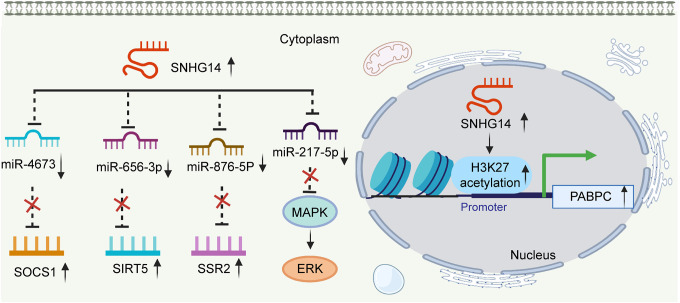
SNHG14 mediates mechanisms involved in HCC.

HCC is one of the most commonly diagnosed cancers and ranks the fourth leading cause of cancer-related deaths worldwide in 2018 (Bray et al., 2018). Dysregulated lncRNAs have been found closely related to tumorigenesis, prognosis, and diagnosis (Abbastabar et al., 2018; Wong et al., 2018; Pan et al., 2019). SNHG14 was found highly expressed in HCC cell lines compared with that in the normal cell line. As for biological function, the overexpression of this lncRNA accelerated cell proliferation, invasion, and migration and suppressed cell apoptosis. Conversely, knockdown of SNHG14 could cause the exact opposite effects on HCC cells (Pu et al., 2019; Tang and Yang, 2020; Xu X. et al., 2020; Zhang H. et al., 2020; Liao et al., 2021). Mechanistic investigations demonstrated that SNHG14 functions as a competing endogenous RNA and sponged miRNAs, such as miR-4673, miR-217-5p, miR-656-3p, and miR-876-5p (Pu et al., 2019; Tang and Yang, 2020; Zhang H. et al., 2020; Liao et al., 2021). Thus, activating downstream gene expression, such as, SOCS1, SIRT5, and SSR2 (Figure 2; Pu et al., 2019; Tang and Yang, 2020; Liao et al., 2021). Another research group reported that SNHG14 contributed to tumor cell malignant cells by increasing poly(A) binding protein cytoplasmic 1 (PABPC1) expression through H3K27 acetylation. In this study, gain- and loss-of-function experiments also revealed that the phosphatase and tensin homolog (PTEN) signaling pathway was involved in SNHG14/PABPC1-mediated regulation of tumorigenesis *in vitro* and *in vivo* (Figure 2; Zhang H. et al., 2020). Accordingly, SNHG14 has an oncogenic role and might be a potential therapeutic target in HCC.

### Ovarian Cancer

The SNHG14 expression level was enormously high in ovarian cancer cells compared with the control cell line (Zhao J. L. et al., 2019; Zhao and Huang, 2019). Zhao et al. discovered that overexpressed SNHG14 could accelerate cell proliferation and cell cycle. Dual-luciferase assay indicated that SNHG14 could directly bind to miR-125a-5p, and overexpression of miR-125a-5p reversed the effect of promoting tumor of SNHG14 on ovarian cancer cells (Zhao and Huang, 2019). SNHG14 was also found to promote ovarian cancer metastasis by regulating DGCR8 expression (Zhao J. L. et al., 2019). Although the underlying molecular mechanism and signaling pathway need to be further studied, the aforementioned evidence proved novel clues for the treatment of ovarian cancer.

### Breast Cancer

Breast cancer with overexpression of human epidermal growth factor receptor 2 (HER2) accounted for 20–30% of all breast cancers and had poorer prognosis (Vu and Claret, 2012; Robidoux et al., 2013). Trastuzumab is an HER2 inhibitor that is used for initial and advanced treatment. However, trastuzumab resistance has been a significant obstacle to improving the outcome of patients (Wolff et al., 2007; Narayan et al., 2009). To explore the contributions of lncRNAs in trastuzumab resistance and progression of breast cancer, Dong et al. (2018b) cultured human breast cancer cell lines SKBR-3 and BT474 and trastuzumab-resistant SKBR-3/Tr and BT474/Tr cells to identify the role of SNHG14 in breast cancer progression and drug resistance. Functional experimentation demonstrated that knockdown of SNHG14 restrain cell proliferation, invasion, trastuzumab resistance, and the overexpression of SNHG14 abolished this effect. Consistent with this effect, Dong et al. (2018a) also found SNHG14 promoted trastuzumab chemoresistance in breast cancer. Thus, SNHG14 may serve as a promising target for patients with HER2-positive breast cancer.

### Colorectal Cancer

In colorectal cancer, Di et al. (2019) found that the level of SNHG14 was markedly upregulated in colorectal cancer cell lines compared with that in the control colonic cell line (Wang et al., 2021b). Overexpression of SNHG14 promoted colorectal cancer cell proliferation, invasion, and migration and epithelial-mesenchymal transition *in vitro* and enhanced tumor growth and distant metastasis *in vivo* (Di et al., 2019; Pei et al., 2019; Ye et al., 2019; Han et al., 2020). Furthermore, mechanistic investigations demonstrated that SNHG14 facilitates colorectal cancer progression by targeting EZH2-regulated EPHA7 and absorbing miR-186-5p (Di et al., 2019). Pei et al. (2019) reported that SNHG14 could serve as an oncogene by regulating the miR-944/KRAS axis via the PI3K/AKT signaling pathway. SNHG14 was also found to regulate colorectal cancer progression via the miR-32-5p/SKIL and miR-186/ATG14 axes (Ye et al., 2019; Han et al., 2020). In contrast to the aforementioned study, a single study reported that SNHG14 was significantly down-regulated in colorectal cancer cell lines compared with that in a normal cell line and SNHG14 exerts an anti-tumor effect through sponging miR-92b-3p (Zhang W. et al., 2020). Thus, further studies are still needed before determining conclusions related to the function and regulatory mechanisms of SNHG14 in colorectal cancer.

### Glioma

Lu et al. (2020) revealed that SNHG14 is involved in reprogramming glucose metabolism and tumorigenesis by interacting with RNA-binding protein Lin28A in glioma. Silencing SNHG14 inhibited glioma cell glycolysis and proliferation while enhancing apoptosis. In contrast to the aforementioned study, another study demonstrated the role of SNHG144 in the suppression of cell proliferation and invasion and promotion of apoptosis in glioma (Wang Q. et al., 2018).

### Pancreatic Cancer

**FIGURE 3:**
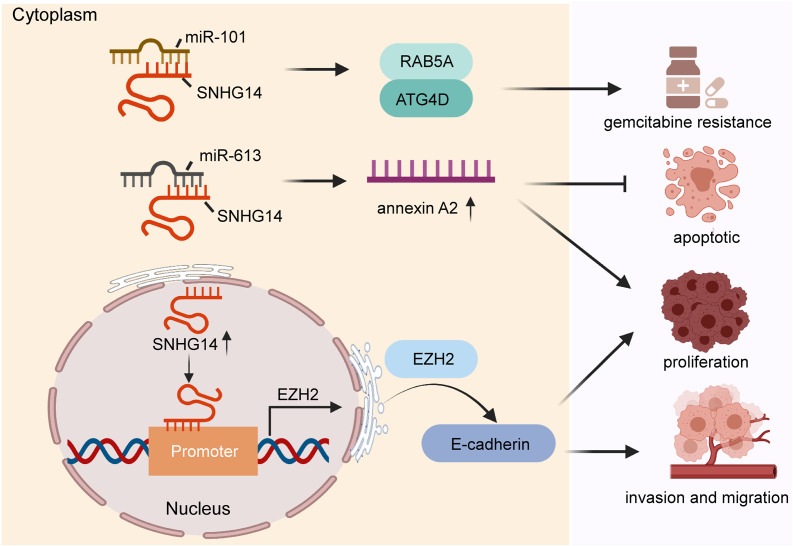
Function and molecular mechanism of SNHG14 in pancreatic cancer.

Pancreatic cancer a highly fatal gastrointestinal malignancy and ranks the seventh leading cause of cancer-related deaths (Luchini et al., 2016). Although the diagnosis and management of pancreatic cancer are improved, the 5-year survival rate is as low as 4% (Vincent et al., 2011; Gandhi et al., 2018). Pancreatic ductal adenocarcinoma is the most common pathological type of pancreatic cancer and lacks effective treatment (Luchini et al., 2016; Gallmeier and Gress, 2018). In recent years, growing amount of evidence shows that lncRNAs may play vital roles in the development and maintenance of pancreatic cancer (Taucher et al., 2016; Moschovis et al., 2017; Wang J. et al., 2020). Some studies have reported that SNHG14 expression was significantly higher in pancreatic cancer cells compared with that in normal cell lines, and upregulated this lncRNA enhanced cell proliferation and invasion through regulating E-cadherin expression via binding on promoters of EZH2 (Xie et al., 2020). Mechanistically, this lncRNA was also found to potentiate tumor progression through modulation of target gene annexin A2 via sponging miR-613 (Deng et al., 2019). Alternatively, Zhang et al. (2019b) reported that SNHG14 increased gemcitabine resistance to pancreatic cancer cells by increasing autophagy-related proteins (such as RAB5A and ATG4D) through interacting with miR-101. The function and molecular mechanism of SNHG14 are shown in Figure 3. Collectively, SNHG14 shows its role in the initiation, progression, and drug resistance, suggesting its potential role in tumor treatment of pancreatic cancer.

### Others

Other studies on cervical cancer (Ji et al., 2019; Zhang et al., 2019c), prostate cancer (Luo et al., 2020), osteosarcoma (Hou and Mao, 2020), endometrial cancer (Zhao et al., 2020), retinoblastoma (Sun et al., 2020), clear cell renal cell carcinoma (Liu et al., 2017), diffuse large B cell lymphoma (Zhao L. et al., 2019; Tian et al., 2021), and acute myeloid leukemia (Wang et al., 2021a) indicated a tumor promoter role for SNHG14 through complex molecular mechanisms. Table 2 summarizes the current results of the function, target genes, and signaling pathways in various malignancies.

## Conclusion and Perspectives

With the development of RNA-seq technologies, bulk lncRNAs are being identified and characterized (Jathar et al., 2017; Wang J. et al., 2018; Qian et al., 2019; Gu et al., 2021). Dysregulation in the expression of lncRNAs has been demonstrated to participate in diverse diseases, especially cancer (Poliseno et al., 2015; Peng and Croce, 2016; Kristensen et al., 2018). LncRNA SNHG14 has been found to be overexpressed in various cancer tissues and is closely associated with multiple clinicopathological characteristics such as prognosis, tumor differentiation, TNM stage, and lymph node metastasis. As a tumor promotion gene, the results of functional experiments have demonstrated that overexpression of this lncRNA could promote tumor cell proliferation, migration, invasion, and chemoresistance and inhibit cell apoptosis. The regulatory mechanism of SNHG14 is complex in distinct types of cancers. To regulate target genes via competing with miRNAs is the major mechanism underlying the tumor-promoting function of SNHG14 by regulating target genes via sponging different miRNAs, such as miR-5590-3p, miR-152-3p, miR-193b-3p, miR-92b-3p, miR-186, miR-32, and miR-93-5p (Table 2).

As for clinical application, high expression of SNHG14 was closely correlated with poorer clinicopathological characteristics; thus, it may be served as a potential biomarker for diagnosis and prognosis. However, most of the studies lack a sufficiently large sample and might have artificial errors. The expression of SNHG14 was tested only in tissues. The expression level of SNHG14 in serum or other biological samples remains unclear and is worth investigating. Therefore, exploring the expression of SNHG14 in blood and other fluids would be beneficial to its clinical application as a diagnostic marker in the future. Additionally, although several studies have addressed the promising role of SNHG14 as a target for cancer treatment, research on SNHG14 is still in its early stages. When could targeting SNHG14 be used in clinical treatment? The main determinant is the availability of drugs (whether oligonucleotide or small molecule drugs) that manipulate SNHG14 activity and deliver them effectively to tumor cells with lasting effects. Furthermore, clarification of the functions and mechanisms of SNHG14 under physiological and pathological conditions is also necessary.

## Author Contributions

SS and YW wrote and reviewed the manuscript. YZ collected the references. ZD and JX reviewed the manuscript. All authors contributed to the writing and revision of the manuscript, knew the content of it, and approved its submission.

## Conflict of Interest

The authors declare that the research was conducted in the absence of any commercial or financial relationships that could be construed as a potential conflict of interest.

## Publisher’s Note

All claims expressed in this article are solely those of the authors and do not necessarily represent those of their affiliated organizations, or those of the publisher, the editors and the reviewers. Any product that may be evaluated in this article, or claim that may be made by its manufacturer, is not guaranteed or endorsed by the publisher.

## References

[B1] Abbastabar M. Sarfi M. Golestani A. Khalili E. (2018). LncRNA involvement in hepatocellular carcinoma metastasis and prognosis. *EXCLI J.* 17 900–913. 10.17179/excli2018-1541 30564069 PMC6295623

[B2] Akhade V. S. Pal D. Kanduri C. (2017). Long noncoding RNA: genome organization and mechanism of action. *Adv. Exp. Med. Biol.* 1008 47–74. 10.1007/978-981-10-5203-3_2 28815536

[B3] Aznaourova M. Schmerer N. Schmeck B. Schulte L. N. (2020). Disease-causing mutations and rearrangements in long non-coding RNA gene loci. *Front. Genet.* 11:527484. 10.3389/fgene.2020.527484 33329688 PMC7735109

[B4] Barlow D. P. Bartolomei M. S. (2014). Genomic imprinting in mammals. *Cold Spring Harb. Perspect. Biol.* 6:a018382. 10.1101/cshperspect.a018382 24492710 PMC3941233

[B5] Bhan A. Soleimani M. Mandal S. S. (2017). Long noncoding RNA and cancer: a new paradigm. *Cancer Res.* 77 3965–3981. 10.1158/0008-5472.Can-16-2634 28701486 PMC8330958

[B6] Bray F. Ferlay J. Soerjomataram I. Siegel R. L. Torre L. A. Jemal A. (2018). Global cancer statistics 2018: globocan estimates of incidence and mortality worldwide for 36 cancers in 185 countries. *CA Cancer J. Clin.* 68 394–424. 10.3322/caac.21492 30207593

[B7] Cao Y. Tian T. Li W. Xu H. Zhan C. Wu X. (2020). Long non-coding RNA in bladder cancer. *Clin. Chim. Acta* 503 113–121. 10.1016/j.cca.2020.01.008 31940466

[B8] Chan J. J. Tay Y. (2018). Noncoding RNA:RNA regulatory networks in cancer. *Int. J. Mol. Sci.* 19:1310. 10.3390/ijms19051310 29702599 PMC5983611

[B9] Chen X. Song P. Yao Y. Yang Y. (2020). Long non-coding rna snhg14 regulates spin1 expression to accelerate tumor progression in non-small cell lung cancer by sponging mir-382-5p. *Cancer Manag. Res.* 12 9113–9123. 10.2147/cmar.S250893 33061605 PMC7524175

[B10] Corona-Gomez J. A. Garcia-Lopez I. J. Stadler P. F. Fernandez-Valverde S. L. (2020). Splicing conservation signals in plant long noncoding RNAs. *RNA* 26 784–793. 10.1261/rna.074393.119 32241834 PMC7297117

[B11] DeGeorge K. C. Holt H. R. Hodges S. C. (2017). Bladder cancer: diagnosis and treatment. *Am. Fam. Phys.* 96 507–514. 29094888

[B12] Deng P. C. Chen W. B. Cai H. H. An Y. Wu X. Q. Chen X. M. (2019). LncRNA snhg14 potentiates pancreatic cancer progression via modulation of annexin a2 expression by acting as a competing endogenous RNA for mir-613. *J. Cell. Mol. Med.* 23 7222–7232. 10.1111/jcmm.14467 31513352 PMC6815841

[B13] Di W. Weinan X. Xin L. Zhiwei Y. Xinyue G. Jinxue T. (2019). Long noncoding RNA snhg14 facilitates colorectal cancer metastasis through targeting ezh2-regulated epha7. *Cell Death Dis.* 10:514. 10.1038/s41419-019-1707-x 31273190 PMC6609685

[B14] Dong H. Wang W. Chen R. Zhang Y. Zou K. Ye M. (2018a). Exosome-mediated transfer of lncRNA-snhg14 promotes trastuzumab chemoresistance in breast cancer. *Int. J. Oncol.* 53 1013–1026. 10.3892/ijo.2018.4467 30015837 PMC6065402

[B15] Dong H. Wang W. Mo S. Liu Q. Chen X. Chen R. (2018b). Long non-coding RNA snhg14 induces trastuzumab resistance of breast cancer via regulating pabpc1 expression through h3k27 acetylation. *J. Cell. Mol. Med.* 22 4935–4947. 10.1111/jcmm.13758 30063126 PMC6156344

[B16] Feng R. Li Z. Wang X. Ge G. Jia Y. Wu D. (2021). Silenced lncRNA snhg14 restrains the biological behaviors of bladder cancer cells via regulating microrna-211-3p/esm1 axis. *Cancer Cell Int.* 21:67. 10.1186/s12935-020-01717-7 33482820 PMC7821404

[B17] Gallmeier E. Gress T. M. (2018). [pancreatic ductal adenocarcinoma]. *Internist* 59 805–822. 10.1007/s00108-018-0460-z 29980819

[B18] Gandhi N. S. Feldman M. K. Le O. Morris-Stiff G. (2018). Imaging mimics of pancreatic ductal adenocarcinoma. *Abdom. Radiol.* 43 273–284. 10.1007/s00261-017-1330-1 29038855

[B19] Ghafouri-Fard S. Dashti S. Taheri M. Omrani M. D. (2020a). Tincr: an lncRNA with dual functions in the carcinogenesis process. *Noncoding RNA Res.* 5 109–115. 10.1016/j.ncrna.2020.06.003 32695943 PMC7358216

[B20] Ghafouri-Fard S. Omrani M. D. Taheri M. (2020b). Long noncoding RNA pvt1: a highly dysregulated gene in malignancy. *J. Cell. Physiol.* 235 818–835. 10.1002/jcp.29060 31297833

[B21] Ginn L. Shi L. Montagna M. Garofalo M. (2020). LncRNAs in non-small-cell lung cancer. *Noncoding RNA* 6:25. 10.3390/ncrna6030025 32629922 PMC7549371

[B22] Gu X. Chu Q. Zheng Q. Wang J. Zhu H. (2021). The dual functions of the long noncoding RNA casc15 in malignancy. *Biomed. Pharmacother.* 135:111212. 10.1016/j.biopha.2020.111212 33433353

[B23] Han P. Chang C. P. (2015). Long non-coding RNA and chromatin remodeling. *RNA Biol.* 12 1094–1098. 10.1080/15476286.2015.1063770 26177256 PMC4829272

[B24] Han Y. Zhou S. Wang X. Mao E. Huang L. (2020). Snhg14 stimulates cell autophagy to facilitate cisplatin resistance of colorectal cancer by regulating mir-186/atg14 axis. *Biomed. Pharmacother.* 121:109580. 10.1016/j.biopha.2019.109580 31704614

[B25] Hou X. K. Mao J. S. (2020). Long noncoding RNA snhg14 promotes osteosarcoma progression via mir-433-3p/fbxo22 axis. *Biochem. Biophys. Res. Commun.* 523 766–772. 10.1016/j.bbrc.2020.01.016 31948764

[B26] Huarte M. (2015). The emerging role of lncRNAs in cancer. *Nat. Med.* 21 1253–1261. 10.1038/nm.3981 26540387

[B27] Jathar S. Kumar V. Srivastava J. Tripathi V. (2017). Technological developments in lncRNA biology. *Adv. Exp. Med. Biol.* 1008 283–323. 10.1007/978-981-10-5203-3_10 28815544

[B28] Ji N. Wang Y. Bao G. Yan J. Ji S. (2019). LncRNA snhg14 promotes the progression of cervical cancer by regulating mir-206/ywhaz. *Pathol. Res. Pract.* 215 668–675. 10.1016/j.prp.2018.12.026 30611620

[B29] Jiang H. Ni J. Zheng Y. Xu Y. (2021). Knockdown of lncRNA snhg14 alleviates lps-induced inflammation and apoptosis of pc12 cells by regulating mir-181b-5p. *Exp. Ther. Med.* 21:497. 10.3892/etm.2021.9928 33791006 PMC8005701

[B30] Jiao P. Hou J. Yao M. Wu J. Ren G. (2019). Snhg14 silencing suppresses the progression and promotes cisplatin sensitivity in non-small cell lung cancer. *Biomed. Pharmacother.* 117:109164. 10.1016/j.biopha.2019.109164 31252267

[B31] Ko E. C. Raben D. Formenti S. C. (2018). The integration of radiotherapy with immunotherapy for the treatment of non-small cell lung cancer. *Clin. Cancer Res.* 24 5792–5806. 10.1158/1078-0432.Ccr-17-3620 29945993

[B32] Kristensen L. S. Hansen T. B. Venø M. T. Kjems J. (2018). Circular RNAs in cancer: opportunities and challenges in the field. *Oncogene* 37 555–565. 10.1038/onc.2017.361 28991235 PMC5799710

[B33] Lenis A. T. Lec P. M. Chamie K. Mshs M. D. (2020). Bladder cancer: a review. *JAMA* 324 1980–1991. 10.1001/jama.2020.17598 33201207

[B34] Li J. Wang A. S. Wang S. Wang C. Y. Xue S. Guan H. (2019). Lncsnhg14 promotes the development and progression of bladder cancer by targeting mirna-150-5p. *Eur. Rev. Med. Pharmacol. Sci.* 23 1022–1029. 10.26355/eurrev_201902_16989 30779068

[B35] Li Y. Li G. Guo X. Yao H. Wang G. Li C. (2020). Non-coding RNA in bladder cancer. *Cancer Lett.* 485 38–44. 10.1016/j.canlet.2020.04.023 32437725

[B36] Liao Z. Zhang H. Su C. Liu F. Liu Y. Song J. (2021). Long noncoding RNA snhg14 promotes hepatocellular carcinoma progression by regulating mir-876-5p/ssr2 axis. *J. Exp. Clin. Cancer Res.* 40:36. 10.1186/s13046-021-01838-5 33485374 PMC7824933

[B37] Lin R. X. Zhan G. F. Wu J. C. Fang H. Yang S. L. (2021). LncRNA snhg14 sponges mir-206 to affect proliferation, apoptosis, and metastasis of hepatocellular carcinoma cells by regulating sox9. *Dig. Dis. Sci.* 10.1007/s10620-021-06920-8 [Epub ahead of print]. 33782806

[B38] Liu G. Ye Z. Zhao X. Ji Z. (2017). Sp1-induced up-regulation of lncRNA snhg14 as a ceRNA promotes migration and invasion of clear cell renal cell carcinoma by regulating n-wasp. *Am. J. Cancer Res.* 7 2515–2525. 29312804 PMC5752691

[B39] Lu J. Liu X. Zheng J. Song J. Liu Y. Ruan X. (2020). Lin28a promotes irf6-regulated aerobic glycolysis in glioma cells by stabilizing snhg14. *Cell Death Dis.* 11:447. 10.1038/s41419-020-2650-6 32527996 PMC7289837

[B40] Luchini C. Capelli P. Scarpa A. (2016). Pancreatic ductal adenocarcinoma and its variants. *Surg. Pathol. Clin.* 9 547–560. 10.1016/j.path.2016.05.003 27926359

[B41] Luo Z. F. Peng Y. Liu F. H. Ma J. S. Hu G. Lai S. L. (2020). Long noncoding RNA snhg14 promotes malignancy of prostate cancer by regulating with mir-5590-3p/yy1 axis. *Eur. Rev. Med. Pharmacol. Sci.* 24 4697–4709. 10.26355/eurrev_202005_21158 32432733

[B42] Martens-Uzunova E. S. Böttcher R. Croce C. M. Jenster G. Visakorpi T. Calin G. A. (2014). Long noncoding RNA in prostate, bladder, and kidney cancer. *Eur. Urol.* 65 1140–1151. 10.1016/j.eururo.2013.12.003 24373479

[B43] Moschovis D. Gazouli M. Tzouvala M. Vezakis A. Karamanolis G. (2017). Long non-coding RNA in pancreatic adenocarcinoma and pancreatic neuroendocrine tumors. *Ann. Gastroenterol.* 30 622–628. 10.20524/aog.2017.0185 29118556 PMC5670281

[B44] Narayan M. Wilken J. A. Harris L. N. Baron A. T. Kimbler K. D. Maihle N. J. (2009). Trastuzumab-induced her reprogramming in “resistant” breast carcinoma cells. *Cancer Res.* 69 2191–2194. 10.1158/0008-5472.Can-08-1056 19276389

[B45] Ørom U. A. Derrien T. Beringer M. Gumireddy K. Gardini A. Bussotti G. (2010). Long noncoding RNAs with enhancer-like function in human cells. *Cell* 143 46–58. 10.1016/j.cell.2010.09.001 20887892 PMC4108080

[B46] Pan W. Li W. Zhao J. Huang Z. Zhao J. Chen S. (2019). LncRNA-pdpk2p promotes hepatocellular carcinoma progression through the pdk1/akt/caspase 3 pathway. *Mol. Oncol.* 13 2246–2258. 10.1002/1878-0261.12553 31368655 PMC6763783

[B47] Pei Q. Liu G. S. Li H. P. Zhang Y. Xu X. C. Gao H. (2019). Long noncoding RNA snhg14 accelerates cell proliferation, migration, invasion and suppresses apoptosis in colorectal cancer cells by targeting mir-944/kras axis through pi3k/akt pathway. *Eur. Rev. Med. Pharmacol. Sci.* 23 9871–9881. 10.26355/eurrev_201911_19551 31799655

[B48] Peng Y. Croce C. M. (2016). The role of micrornas in human cancer. *Signal Transduct. Target Ther.* 1:15004. 10.1038/sigtrans.2015.4 29263891 PMC5661652

[B49] Poliseno L. Marranci A. Pandolfi P. P. (2015). Pseudogenes in human cancer. *Front. Med.* 2:68. 10.3389/fmed.2015.00068 26442270 PMC4585173

[B50] Pu J. Wei H. Tan C. Qin B. Zhang Y. Wang A. (2019). Long noncoding RNA snhg14 facilitates hepatocellular carcinoma progression through regulating mir-4673/socs1. *Am. J. Transl. Res.* 11 5897–5904. 31632558 PMC6789279

[B51] Qi X. Shao M. Sun H. Shen Y. Meng D. Huo W. (2017). Long non-coding rna snhg14 promotes microglia activation by regulating mir-145-5p/pla2g4a in cerebral infarction. *Neuroscience* 348 98–106. 10.1016/j.neuroscience.2017.02.002 28215748

[B52] Qian X. Zhao J. Yeung P. Y. Zhang Q. C. Kwok C. K. (2019). Revealing lncRNA structures and interactions by sequencing-based approaches. *Trends Biochem. Sci.* 44 33–52. 10.1016/j.tibs.2018.09.012 30459069

[B53] Robidoux A. Tang G. Rastogi P. Geyer C. E. Jr. Azar C. A. Atkins J. N. (2013). Lapatinib as a component of neoadjuvant therapy for her2-positive operable breast cancer (nsabp protocol b-41): an open-label, randomised phase 3 trial. *Lancet Oncol.* 14 1183–1192. 10.1016/s1470-2045(13)70411-x 24095300

[B54] Romero-Barrios N. Legascue M. F. Benhamed M. Ariel F. Crespi M. (2018). Splicing regulation by long noncoding RNAs. *Nucleic Acids Res.* 46 2169–2184. 10.1093/nar/gky095 29425321 PMC5861421

[B55] Schmitz S. U. Grote P. Herrmann B. G. (2016). Mechanisms of long noncoding RNA function in development and disease. *Cell. Mol. Life Sci.* 73 2491–2509. 10.1007/s00018-016-2174-5 27007508 PMC4894931

[B56] Shi C. Zhao Y. Li Q. Li J. (2021). LncRNA snhg14 plays a role in sepsis-induced acute kidney injury by regulating mir-93. *Mediators Inflamm.* 2021:5318369. 10.1155/2021/5318369 33505213 PMC7806393

[B57] Simion V. Haemmig S. Feinberg M. W. (2019). LncRNAs in vascular biology and disease. *Vascul. Pharmacol.* 114 145–156. 10.1016/j.vph.2018.01.003 29425892 PMC6078824

[B58] Statello L. Guo C. J. Chen L. L. Huarte M. (2021). Gene regulation by long non-coding RNAs and its biological functions. *Nat. Rev. Mol. Cell Biol.* 22 96–118. 10.1038/s41580-020-00315-9 33353982 PMC7754182

[B59] Sun X. Shen H. Liu S. Gao J. Zhang S. (2020). Long noncoding RNA snhg14 promotes the aggressiveness of retinoblastoma by sponging microrna-124 and thereby upregulating stat3. *Int. J. Mol. Med.* 45 1685–1696. 10.3892/ijmm.2020.4547 32236565 PMC7169960

[B60] Tang S. J. Yang J. B. (2020). LncRNa snhg14 aggravates invasion and migration as ceRNA via regulating mir-656-3p/sirt5 pathway in hepatocellular carcinoma. *Mol. Cell. Biochem.* 473 143–153. 10.1007/s11010-020-03815-6 32607966

[B61] Taucher V. Mangge H. Haybaeck J. (2016). Non-coding RNAs in pancreatic cancer: challenges and opportunities for clinical application. *Cell. Oncol.* 39 295–318. 10.1007/s13402-016-0275-7 27060060 PMC13001889

[B62] Tian D. Sun S. Lee J. T. (2010). The long noncoding RNA, jpx, is a molecular switch for x chromosome inactivation. *Cell* 143 390–403. 10.1016/j.cell.2010.09.049 21029862 PMC2994261

[B63] Tian Y. Li L. Lin G. Wang Y. Wang L. Zhao Q. (2021). lncRNA SNHG14 promotes oncogenesis and immune evasion in diffuse large-B-cell lymphoma by sequestering miR-152-3p. *Leukemia Lymphoma* 62 1574–1584. 10.1080/10428194.2021.1876866 33682607

[B64] Vincent A. Herman J. Schulick R. Hruban R. H. Goggins M. (2011). Pancreatic cancer. *Lancet* 378 607–620. 10.1016/s0140-6736(10)62307-0 21620466 PMC3062508

[B65] Vu T. Claret F. X. (2012). Trastuzumab: updated mechanisms of action and resistance in breast cancer. *Front. Oncol.* 2:62. 10.3389/fonc.2012.00062 22720269 PMC3376449

[B66] Wang J. Su Z. Lu S. Fu W. Liu Z. Jiang X. (2018). LncRNA hoxa-as2 and its molecular mechanisms in human cancer. *Clin. Chim. Acta* 485 229–233. 10.1016/j.cca.2018.07.004 29981289

[B67] Wang J. Zhao L. Shang K. Liu F. Che J. Li H. (2020). Long non-coding RNA h19, a novel therapeutic target for pancreatic cancer. *Mol. Med.* 26:30. 10.1186/s10020-020-00156-4 32272875 PMC7146949

[B68] Wang Q. Teng Y. Wang R. Deng D. You Y. Peng Y. (2018). The long non-coding RNA snhg14 inhibits cell proliferation and invasion and promotes apoptosis by sponging mir-92a-3p in glioma. *Oncotarget* 9 12112–12124. 10.18632/oncotarget.23960 29552296 PMC5844732

[B69] Wang X. Li W. Chen Y. Zhou L. (2021a). Long non-coding RNA snhg14 affects the proliferation and apoptosis of childhood acute myeloid leukaemia cells by modulating the mir-193b-3p/mcl1 axis. *Mol. Med. Rep.* 23:90. 10.3892/mmr.2020.11729 33300066

[B70] Wang X. Yang P. Zhang D. Lu M. Zhang C. Sun Y. (2021b). LncRNA snhg14 promotes cell proliferation and invasion in colorectal cancer through modulating mir-519b-3p/ddx5 axis. *J. Cancer* 12 4958–4970. 10.7150/jca.55495 34234865 PMC8247390

[B71] Winkle M. El-Daly S. M. Fabbri M. Calin G. A. (2021). Noncoding rna therapeutics - challenges and potential solutions. *Nat. Rev. Drug Discov.* 20 629–651. 10.1038/s41573-021-00219-z 34145432 PMC8212082

[B72] Wolff A. C. Hammond M. E. Schwartz J. N. Hagerty K. L. Allred D. C. Cote R. J. (2007). American society of clinical oncology/college of american pathologists guideline recommendations for human epidermal growth factor receptor 2 testing in breast cancer. *J. Clin. Oncol.* 25 118–145. 10.1200/jco.2006.09.2775 17159189

[B73] Wong C. M. Tsang F. H. Ng I. O. (2018). Non-coding RNAs in hepatocellular carcinoma: molecular functions and pathological implications. *Nat. Rev. Gastroenterol. Hepatol.* 15 137–151. 10.1038/nrgastro.2017.169 29317776

[B74] Wu K. Li J. Qi Y. Zhang C. Zhu D. Liu D. (2019). Snhg14 confers gefitinib resistance in non-small cell lung cancer by up-regulating abcb1 via sponging mir-206-3p. *Biomed. Pharmacother.* 116:108995. 10.1016/j.biopha.2019.108995 31121484

[B75] Xie F. Huang Q. Wang C. Chen S. Liu C. Lin X. (2020). Downregulation of long noncoding RNA snhg14 suppresses cell proliferation and invasion by regulating ezh2 in pancreatic ductal adenocarcinoma (pdac). *Cancer Biomark.* 27 357–364. 10.3233/cbm-190908 31929143 PMC12662296

[B76] Xu L. Xu Y. Yang M. Li J. Xu F. Chen B. L. (2020). LncRNA snhg14 regulates the ddp-resistance of non-small cell lung cancer cell through mir-133a/hoxb13 pathway. *BMC Pulm. Med.* 20:266. 10.1186/s12890-020-01276-7 33059643 PMC7559791

[B77] Xu X. Song F. Jiang X. Hong H. Fei Q. Jin Z. (2020). Long non-coding RNA snhg14 contributes to the development of hepatocellular carcinoma via sponging mir-217. *Onco Targets Ther.* 13 4865–4876. 10.2147/ott.S244530 32581548 PMC7269013

[B78] Yang N. Wang H. Zhang L. Lv J. Niu Z. Liu J. (2021). Long non-coding RNA snhg14 aggravates lps-induced acute kidney injury through regulating mir-495-3p/hipk1. *Acta Biochim. Biophys. Sin.* 53 719–728. 10.1093/abbs/gmab034 33856026

[B79] Yarani R. Mirza A. H. Kaur S. Pociot F. (2018). The emerging role of lncRNAs in inflammatory bowel disease. *Exp. Mol. Med.* 50 1–14. 10.1038/s12276-018-0188-9 30523244 PMC6283835

[B80] Ye T. Zhang N. Wu W. Yang B. Wang J. Huang W. (2019). Snhg14 promotes the tumorigenesis and metastasis of colorectal cancer through mir-32-5p/skil axis. *In Vitro Cell. Dev. Biol. Anim.* 55 812–820. 10.1007/s11626-019-00398-5 31471872

[B81] Zhang H. Xu H. B. Kurban E. Luo H. W. (2020). LncRNA snhg14 promotes hepatocellular carcinoma progression via h3k27 acetylation activated pabpc1 by pten signaling. *Cell Death Dis.* 11:646. 10.1038/s41419-020-02808-z 32811821 PMC7434898

[B82] Zhang K. Cai Y. Zhou Q. Sun H. Wei J. (2020). Long non-coding RNA snhg14 impedes viability, migration and invasion of endometrial carcinoma cells through modulating mir-93-5p/zbtb7a axis. *Cancer Manag. Res.* 12 9515–9525. 10.2147/cmar.S257419 33061638 PMC7534865

[B83] Zhang W. Duan W. Mo Z. Wang J. Yang W. Wu W. (2020). Upregulation of snhg14 suppresses cell proliferation and metastasis of colorectal cancer by targeting mir-92b-3p. *J. Cell. Biochem.* 121 1998–2008. 10.1002/jcb.29434 31692034

[B84] Zhang X. Wang W. Zhu W. Dong J. Cheng Y. Yin Z. (2019a). Mechanisms and functions of long non-coding RNAs at multiple regulatory levels. *Int. J. Mol. Sci.* 20:5573. 10.3390/ijms20225573 31717266 PMC6888083

[B85] Zhang X. Zhao P. Wang C. Xin B. (2019b). Snhg14 enhances gemcitabine resistance by sponging mir-101 to stimulate cell autophagy in pancreatic cancer. *Biochem. Biophys. Res. Commun.* 510 508–514. 10.1016/j.bbrc.2019.01.109 30737032

[B86] Zhang Y. Tang L. (2018). The application of lncRNAs in cancer treatment and diagnosis. *Recent Pat. Anticancer Drug Discov.* 13 292–301. 10.2174/1574892813666180226121819 29485010

[B87] Zhang Y. Y. Li M. Xu Y. D. Shang J. (2019c). LncRNA snhg14 promotes the development of cervical cancer and predicts poor prognosis. *Eur. Rev. Med. Pharmacol. Sci.* 23 3664–3671. 10.26355/eurrev_201905_17790 31114991

[B88] Zhang Z. Wang Y. Zhang W. Li J. Liu W. Lu W. (2019d). Long non-coding RNA snhg14 exerts oncogenic functions in non-small cell lung cancer through acting as an mir-340 sponge. *Biosci. Rep.* 39:BSR20180941. 10.1042/bsr20180941 30254102 PMC6328883

[B89] Zhao J. L. Wang C. L. Liu Y. L. Zhang G. Y. (2019). Long noncoding rna snhg14 enhances migration and invasion of ovarian cancer by upregulating dgcr8. *Eur. Rev. Med. Pharmacol. Sci.* 23 10226–10233. 10.26355/eurrev_201912_19659 31841176

[B90] Zhao L. Liu Y. Zhang J. Liu Y. Qi Q. (2019). LncRNA snhg14/mir-5590-3p/zeb1 positive feedback loop promoted diffuse large b cell lymphoma progression and immune evasion through regulating pd-1/pd-l1 checkpoint. *Cell Death Dis.* 10:731. 10.1038/s41419-019-1886-5 31570691 PMC6769008

[B91] Zhao L. Zhang X. Shi Y. Teng T. (2020). LncRNA snhg14 contributes to the progression of nsclc through mir-206/g6pd pathway. *Thorac. Cancer* 11 1202–1210. 10.1111/1759-7714.13374 32153123 PMC7180566

[B92] Zhao Y. L. Huang Y. M. (2019). Lncsnhg14 promotes ovarian cancer by targeting microrna-125a-5p. *Eur. Rev. Med. Pharmacol. Sci.* 23 3235–3242. 10.26355/eurrev_201904_17683 31081075

[B93] Zhong Y. Yu C. Qin W. (2019). LncRNA snhg14 promotes inflammatory response induced by cerebral ischemia/reperfusion injury through regulating mir-136-5p/rock1. *Cancer Gene Ther.* 26 234–247. 10.1038/s41417-018-0067-5 30546117 PMC6760557

[B94] Zhu J. Fu H. Wu Y. Zheng X. (2013). Function of lncRNAs and approaches to lncRNA-protein interactions. *Sci. China Life Sci.* 56 876–885. 10.1007/s11427-013-4553-6 24091684

